# Tyrosine kinase inhibitors in HER2‐positive breast cancer brain metastases: A systematic review and meta‐analysis

**DOI:** 10.1002/cam4.6180

**Published:** 2023-05-31

**Authors:** Yushuai Yu, Kaiyan Huang, Yuxiang Lin, Jie Zhang, Chuangui Song

**Affiliations:** ^1^ Department of Breast Surgery Fujian Medical University Union Hospital Fuzhou China; ^2^ Breast Cancer Institute, Fujian Medical University Fuzhou China; ^3^ Department of General Surgery Fujian Medical University Union Hospital Fuzhou China

**Keywords:** Brain metastases, breast cancer, HER2‐positive, meta‐analysis, small‐molecule tyrosine kinase inhibitors

## Abstract

**Background:**

Small tyrosine kinase inhibitors (TKIs) show activity against breast cancer brain metastases (BCBM) of the human epidermal growth factor receptor 2 (HER2)‐positive subtype. This meta‐analysis aimed to objectively explore the efficacy and safety of TKIs.

**Methods:**

Electronic databases were searched for relevant clinical trials. We conducted a pairwise meta‐analysis, pooled analysis, and estimated summary survival curves to compare survival outcomes following TKIs therapy for BCBM patients using Stata version 16.0 or R x64 4.0.5.

**Results:**

Thirteen clinical trials involving 987 HER2‐positive BCBM patients were analyzed. A trend of longer progression‐free survival (PFS) was observed in the TKI‐containing arm compared to the non‐TKI‐containing arm (hazard ratio = 0.64, 95% confidence interval [CI]: 0.35–1.15, *p* = 0.132), although the difference is not statistically significant. Summary survival curves reported the summary median PFS and overall survival were 7.9 months and 12.3 months. Subgroup analysis revealed that TKIs combined with capecitabine (TKI + Cap) regimens resulted in improved survival outcomes. Tucatinib may be more effective in BCBM patients. The main grade 3–5 adverse events (AEs) were diarrhea (22%, 95% CI: 14%–32%), neutropenia (11%, 95% CI: 5%–18%), hepatic toxicity (7%, 95% CI: 1%–16%), and sensory neuropathy (6%, 95% CI: 2%–12%).

**Conclusion:**

TKIs therapy improved the survival outcomes of HER2‐positive BCBM patients, especially when combined with capecitabine and tolerable AEs. We also identified the clinical value of tucatinib, which appears to be the most favorable TKI drug for BCBM patients.

## 1 BACKGROUND

Breast cancer (BC) is the most common cancer in women.^1^ The incidence of BC brain metastasis (BCBM) has increased in recent years. BC is the second most common cause of brain metastases, accounts for 15%–20% of all brain metastases.^2^, ^3^ The human epidermal growth factor receptor 2 (HER2) amplified subtype is often considered to have a higher incidence of BM than other molecular subtypes.^4^, ^5^ The survival rate of BCBM patients is extremely low. The registHER study found that the median overall survival (OS) from the time of metastatic BC (MBC) diagnosis in patients with any central nervous system (CNS) event was significantly lower than that in patients with no CNS events (26.3 months vs. 44.6 months).^6^

The treatment modalities for BCBM include whole‐brain radiation therapy (WBRT), stereotactic radiosurgery (SRS), and surgery. Unfortunately, the time to intracranial progression after these therapies is approximately 6–12 months.^7^, ^8^, ^9^ The European Association of Neuro‐Oncology recommends that surgery be considered in patients with 1–3 intracranial metastases lesions, especially for lesions at least 3 cm in diameter.^10^ Traditionally, WBRT has been recommended for patients with multiple BM; however, it provides disappointing local tumor control and poor clinical survival outcomes. Due to these limitations, SRS using a Gamma Knife, Linac, or Cyberknife has been proposed.^11^ However, SRS may cause cognitive complications including memory impairment, seizures, cognitive disturbance, motor weakness, and ataxia.^12^ Thus, systemic treatment is often combined with local treatment modalities that have poor efficacy.

The use of small‐molecule tyrosine kinase inhibitors (TKIs), which inhibit both HER2 and EGFR targets, may improve patient outcomes.^13^ TKIs bind to the intracellular domains of HER2, block tyrosine phosphorylation and downstream signaling events of ligand binding, and compete with ATP at the cytoplasmic catalytic kinase domain. They also have high blood–brain barrier (BBB) penetrability, which may better control intracranial lesions and produce little neurotoxicity.^14^ Several TKIs, including lapatinib, neratinib, afatinib, pyrotinib, and tucatinib, have become available and are currently under investigation. In the pivotal single‐group phase 2 study named LANDSCAPE, patients were included who had HER2‐positive BCBM and did not have previous WBRT, lapatinib, or capecitabine previously. The combination of lapatinib plus capecitabine improved CNS objective response rate (ORR) (65.9%, 95% CI: 50.1–79.5) compared with lapatinib alone. The results showed that lapatinib plus capecitabine could be recommended as a treatment for patients with CBM; however, it may be at the expense of relatively high toxicity.^15^ New‐generation TKIs, including neratinib, pyrotinib, and tucatinib, are promising agents for the treatment of HER2‐positive BCBM.^16^, ^17^, ^18^ Consequently, the role of TKI in the treatment of BM in HER2‐positive BC patients has been gaining attention.

A debate is ongoing over the use of TKIs for treating HER2‐positive BCBM owing to a lack of sufficient clinical evidence, mainly a preponderance of results from single‐arm clinical trials and existing randomized controlled trials (RCTs) that reported mixed results. Questions have also been raised owing to differences in the baseline characteristics of patients in various clinical trials. In addition, there is no consensus on the optimal combination drug with TKIs and the TKI drug with better efficacy. A complete analysis of all relevant clinical studies is important to provide useful clinical references. Therefore, we conducted this meta‐analysis using the latest data to provide a comprehensive overview of TKIs therapy for HER2‐positive BCBM patients.

## 2 METHODS

Our study was registered on INPLASY (INPLASY202170064) and the details are available at inplasy.com (https://doi.org/10.37766/inplasy2021.7.0064). The protocol for this systematic review and meta‐analysis has not been previously published in peer‐reviewed journals.

### 2.1 Selection criteria

We developed the eligibility and exclusion criteria for this study in advance. All clinical trials (RCTs or single‐arm trials) investigated TKI regimens for the treatment of HER2‐positive BC patients with brain metastases. A subgroup analysis of patients with brain metastases within relevant trials was also performed in our study. Survival or tumor response data were available for all patients. The studies were grouped based on treatment regimens, regardless of active or inactive brain metastases, treatment lines, or prior treatment. Phase II or III clinical trials published as full papers or abstracts if full papers were not available (especially those from the American Society of Clinical Oncology [ASCO], European Society for Medical Oncology [ESMO], and San Antonio Breast Cancer Symposiums [SABCS]) were included in the analysis. Case reports, systematic reviews, and retrospective studies were excluded from the analysis. In case of multiple publications from the same trial, only the most recent publication was included.

### 2.2 Search strategy

This meta‐analysis followed the Preferred Reporting Items for Systematic Reviews and Meta‐Analyses (PRISMA) guidelines. Searches were performed on PubMed, Embase, and Cochrane Central Register of Clinical Trials, utilizing the following keywords: “(breast OR mammary) AND (cancer OR carcinoma OR malignant OR neoplasm OR tumor) AND (HER‐2 OR HER2 OR HER2/neu OR ERBB2 OR human epidermal growth factor receptor 2) AND (positive OR +) AND (brain OR central nervous system) AND (metastasis OR metastases OR metastatic) AND (small‐molecule tyrosine kinase inhibitors OR lapatinib OR neratinib OR afatinib OR tucatinib OR pyrotinib)”, only in English, without any limitations on factors such as time or race. We also assessed the ASCO and ESMO annual meetings and SABCS online articles, and included articles published before January 1, 2023. The references of all studies that met the eligibility criteria were examined for other relevant studies, especially for relevant meta‐analyses.

### 2.3 Data extraction

Two investigators (YSY and JZ) independently extracted the data, and all included items required consensus. The first author, year of publication, trial name, locations, trial phase, number of analyzed participants, therapy regimens, therapy details, Eastern Cooperative Oncology Group Performance Status, and the efficacy endpoints of each eligible trial were recorded. The primary outcome was progression‐free survival (PFS). The secondary outcomes were CNS ORR, median PFS, median OS, and grade 3–5 adverse events (AEs). All data were recorded from the original articles or using the GetData Graph Digitizer if the original statistical curve pictures were acquired from the articles.

### 2.4 Definition of outcomes

Outcome analyses, including PFS, CNS ORR, median PFS, and median OS, were conducted based on the investigator‐assessed response according to RECIST 1.1. The data have also been corrected and are indicated in the Characteristics of the Trials. Neutropenia, vomiting/nausea, diarrhea, stomatitis mucositis, skin and subcutaneous tissue disorders, sensory neuropathy, and hepatic toxicity were considered as the most crucial side effects. Grade 3–5 AEs were calculated using the National Cancer Institute Common Terminology Criteria version 4.0.

### 2.5 Statistical methods

We conducted a pairwise meta‐analysis to evaluate PFS as hazard ratios (HRs) with corresponding 95% confidence intervals (95% CI) between TKI‐containing and non‐TKI‐containing regimens using available data. Owing to limited data, single‐arm trials were also included in our study to better describe the efficacy of TKIs in BCBM patients. Because no HRs or risk ratios could be produced with these single‐arm studies, we conducted them in three steps to better demonstrate their effectiveness. First, we attempted to estimate tumor response and survival results using a pooled analysis from a quantitative perspective. Continuous variables, including median PFS and median OS, are presented as pooled mean values. Dichotomous variables, including CNS ORR and grade 3–5 AEs incidence rates, were presented as pooled effect sizes with 95% CIs. Second, to indicate the analysis of time survived to an outcome event that cannot be reflected in pooled analysis, a R package entitled MetaSurv was used to estimate “summary survival curves” from single‐arm studies' K‐M curves by the product‐limit estimator method, then the summary median survival can be calculated based on the summary survival curve.^19^ All data were extracted using the GetData Graph Digitizer if the original statistical curve pictures were acquired from the articles or supplementary materials. Thirdly, subgroup analysis was performed using pooled analysis or estimating “summary survival curves.” We planned a subgroup analysis for the following subgroups^1^: to explore the treatment effect of TKIs with different combinations of drugs across subgroups, subgroup analysis was performed using the following classification variables: TKIs were used alone (monotherapy), TKIs were combined with capecitabine (TKI + Cap), TKIs were combined with other drugs except capecitabine (TKI + Others)^2^; to explore the treatment effect of different types of TKI drugs including lapatinib, tucatinib, neratinib, and pyrotinib.

Cochran's Q chi‐square test was used to examine the heterogeneity across studies, and inconsistencies were tested using the *I* ^2^ statistic. We used a fixed‐effects model for the analysis if the pooled results showed low heterogeneity (*I* ^2^ ≤ 50%). Conversely, a random‐effects model was used for significant heterogeneity (*I* ^2^ > 50%). Begg's and Egger's tests were used to examine the potential publication bias of the included clinical trials. All statistical analyses were performed using Stata version 16.0 or R x64 4.0.5, and statistical significance was set at *p* < 0.05.

### 2.6 Quality assessment

The study quality of the five included RCTs was assessed using the Cochrane risk of bias tool. Eight single‐arm studies were assessed using the MINORS index scored.^20^ We included clinical trials that could ensure their high quality and integrity owing to the high heterogeneity that may appear in single‐arm meta‐analyses, and a randomized effect model was applied to minimize the bias.

### 2.7 Patient and public involvement

The patients and the public were not involved in the design, conduct, reporting, or dissemination plans of our research.

## 3 RESULTS

### 3.1 Overview of literature search and study characteristics

**FIGURE 1:**
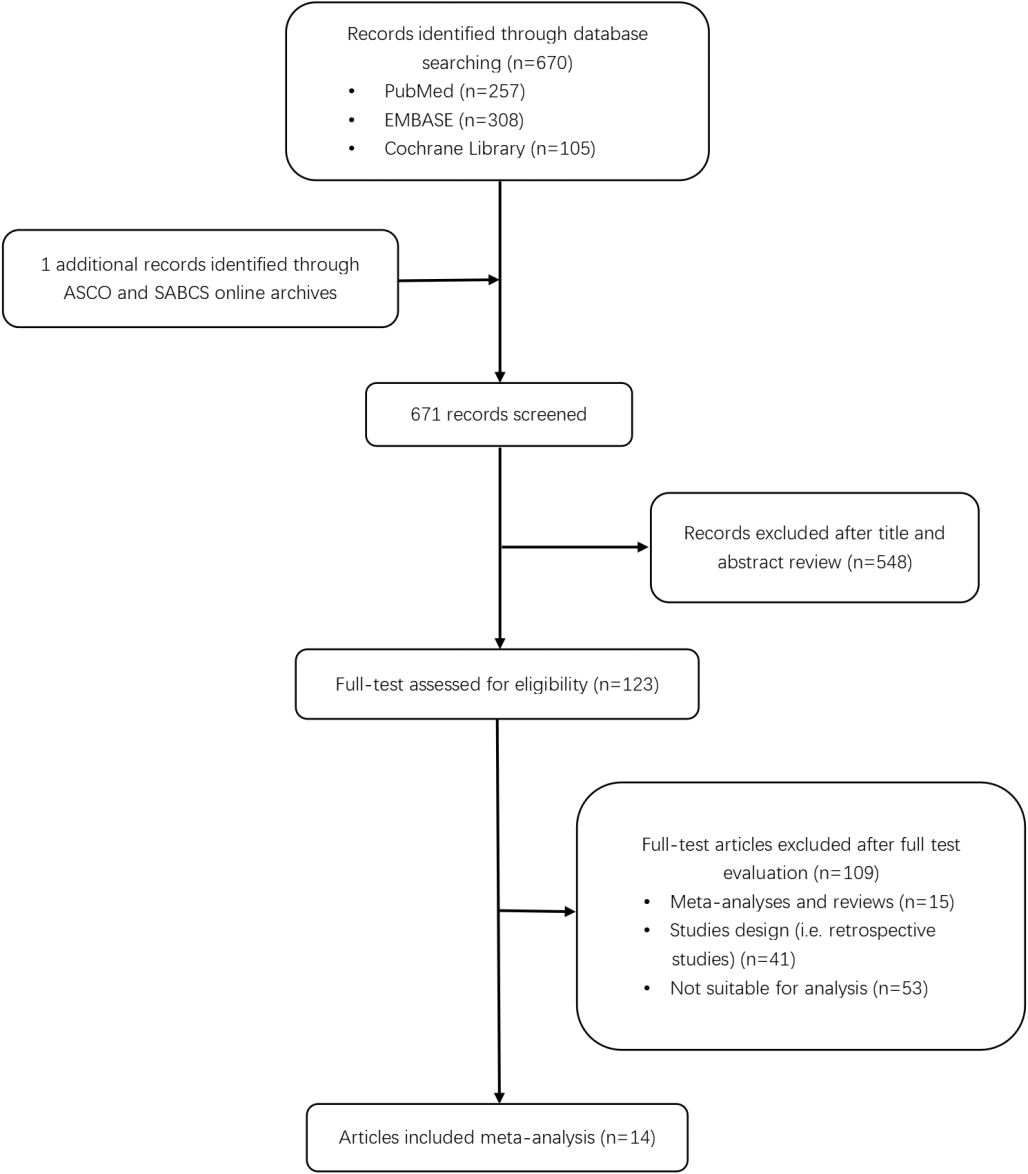
Flowchart diagram of selected studies included in the meta‐analysis.

The initial search revealed 670 potentially relevant manuscripts and one additional abstract. After a detailed review of the titles and abstracts, 548 manuscripts were excluded. The full texts of the remaining 123 articles were read, and a total of 14 articles from 13 trials—including five RCTs and eight single‐arm clinical trials that met the requirements—were obtained according to the inclusion and exclusion criteria (Figure 1).^15^, ^16^, ^17^, ^21^, ^22^, ^23^, ^24^, ^25^, ^26^, ^27^, ^28^, ^29^, ^30^, ^31^ All details of the included trials are described in Table S1. The corresponding PRISMA checklist is shown in Appendix S1. Although the study by Krop et al. was a retrospective study, the results were from a high‐quality multicenter, randomized, open‐label, phase III trial called EMILIA.^21^ Because of the high quality of the study, it was included in our analysis. Among the subgroups, four articles incorporated the results of TKIs for HER2‐positive BCBM, and one article met the eligibility criteria for this meta‐analysis from the abstracts published in ASCO. All eligible studies, which included 987 patients, were published between 2008 and 2022.

### 3.2 Quality assessment

The chart of bias assessment showed a low‐risk bias in all included trials (Appendix S2). Two of the five randomized controlled studies generated detailed random sequences. Because most of the studies (3/5) had open‐label designs, performance bias might have existed, but did not affect the final outcome. All the RCTs provided complete outcome data without bias. However, no allocation concealment or blinding methods have been proposed. The MINORS index score was between 14 and 15 points in eight single studies, which was appropriate for the current study.

### 3.3 Efficacy

#### 3.3.1 Pairwise meta‐analysis for PFS

**FIGURE 2:**
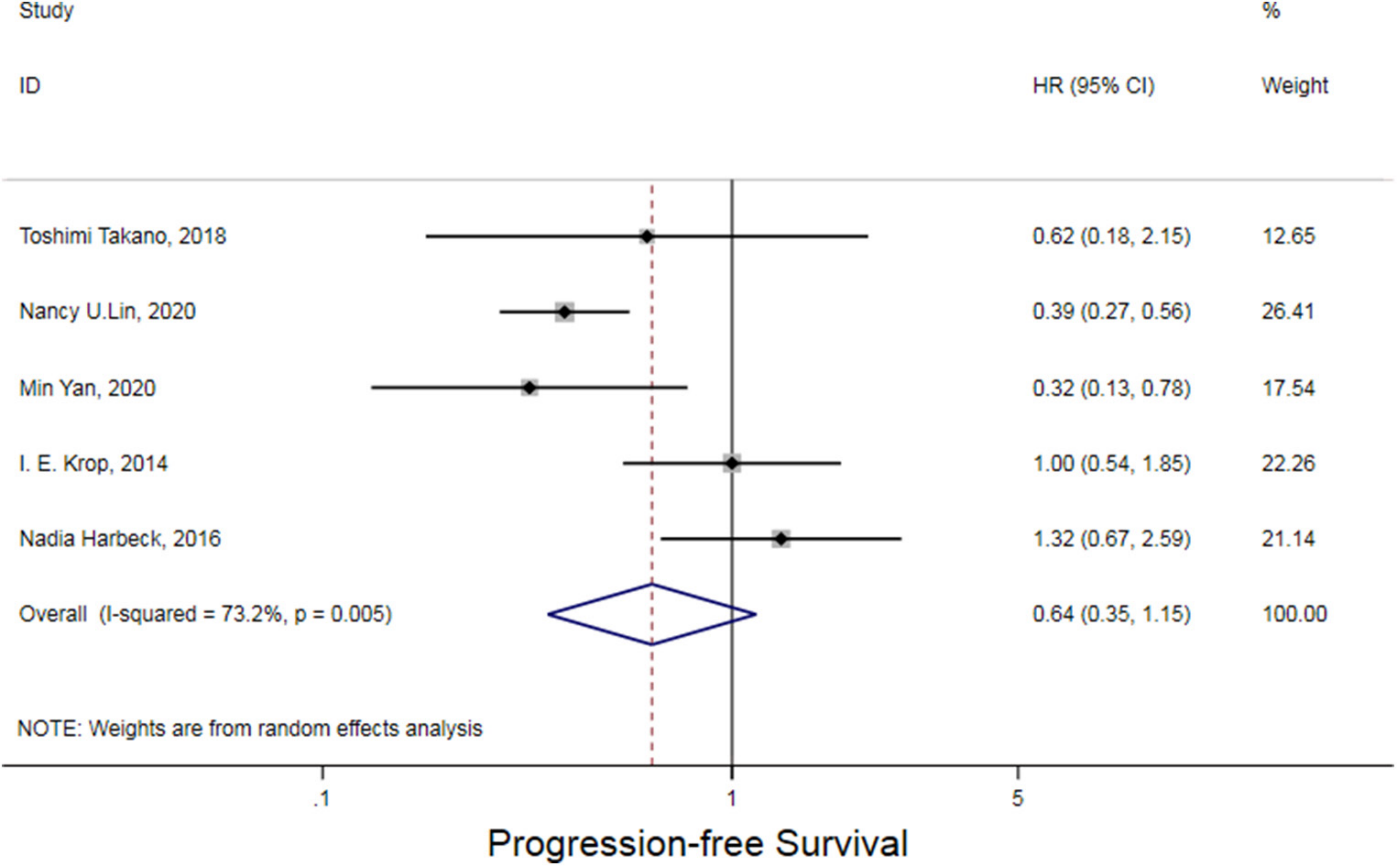
Hazard ratio (HR) of median progression‐free survival (PFS) TKI‐containing regimens for HER2‐positive BCBM patients included.

Five RCTs compared the HRs of PFS compared TKI‐containing regimens with non‐TKI‐containing regimens. The pooled analysis results are provided in Figure 2, which revealed a PFS trend favoring TKI‐containing regimens was observed in BCBM patients but without statistical significance (HR = 0.64, 95% CI: 0.35–1.15; *p* = 0.132) as calculated by the random‐effects model.

#### 3.3.2 Pooled analysis in single‐arm studies

**FIGURE 3:**
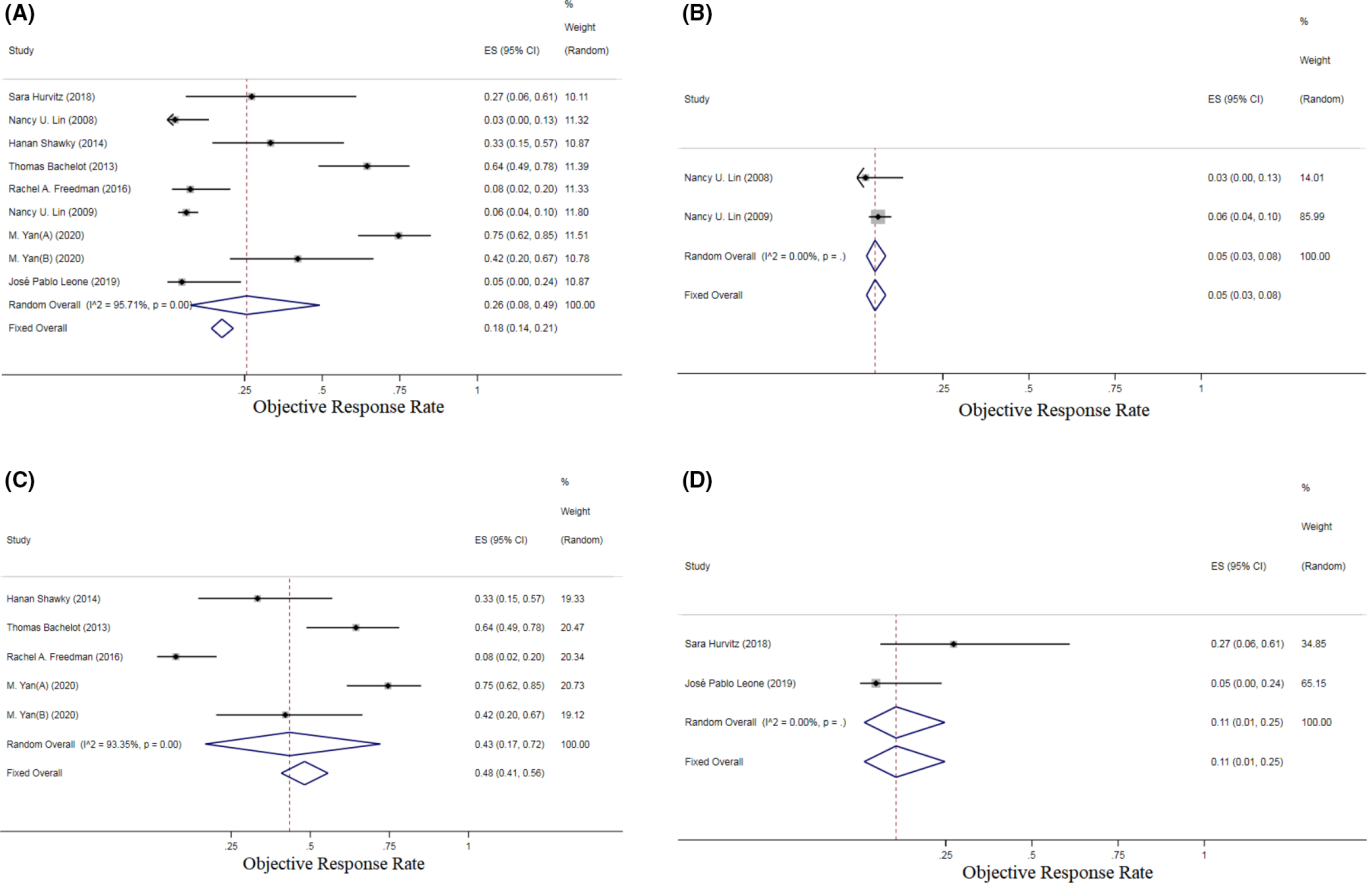
Pooled objective response rate (ORR) in TKI‐containing regimens for HER2‐positive BCBM patients included. (A) TKI‐containing regimens; (B) TKI (Monotherapy); (C) TKI + Cap‐containing regimens; (D) TKI + Other‐containing regimens. Notes: To explore the treatment effect of TKI‐containing regimens with different combinations of drugs across subgroups, subgroup analysis was performed using the following classification variables: TKIs were used alone (monotherapy), TKIs were combined with capecitabine (TKI + Cap), TKIs were combined with other drugs except capecitabine, and they were classified into the TKI + Others group.

CNS ORR data were available for analysis from eight trials and included 497 patients. We noted a wide variation in tumor response in these trials ranged from 3% to 75%. The pooled CNS ORR was 26% (95% CI: 8.0%–49.0%, *I* ^2^ = 95.71%, *p* < 0.001), as calculated using the random‐effects model with high heterogeneity (Figure 3). The treatment effects of TKIs in different drug combinations were explored using subgroup analysis. The pooled results showed significant differences in efficacy between drugs paired with TKIs. The TKI + Cap regimen (43%, 95% CI: 17%–72%) showed a conspicuous CNS ORR advantage over TKI (monotherapy) (5%, 95% CI: 3%–8%) and TKI + Others (11%, 95% CI: 1%–25%) (Figure 3). The pooled median PFS and OS reached 5.72 months (95% CI: 3.54–7.89) and 9.18 months (95% CI: 6.37–11.99), also confirmed that TKI‐containing regimens are promising therapeutic choices for BCBM patients (Figure S1). Funnel plots of the five included RCTs revealed no obvious evidence of publication bias (Figure S2). The pooled results of the median PFS, median OS, and CNS ORR in total, and the CNS ORR of the TKI + Cap, TKI (monotherapy), and TKI + Others regimens were proven to be reliable through Begg's and Egger's tests.

#### 3.3.3 “Summary survival curves” in single‐arm studies

**FIGURE 4:**
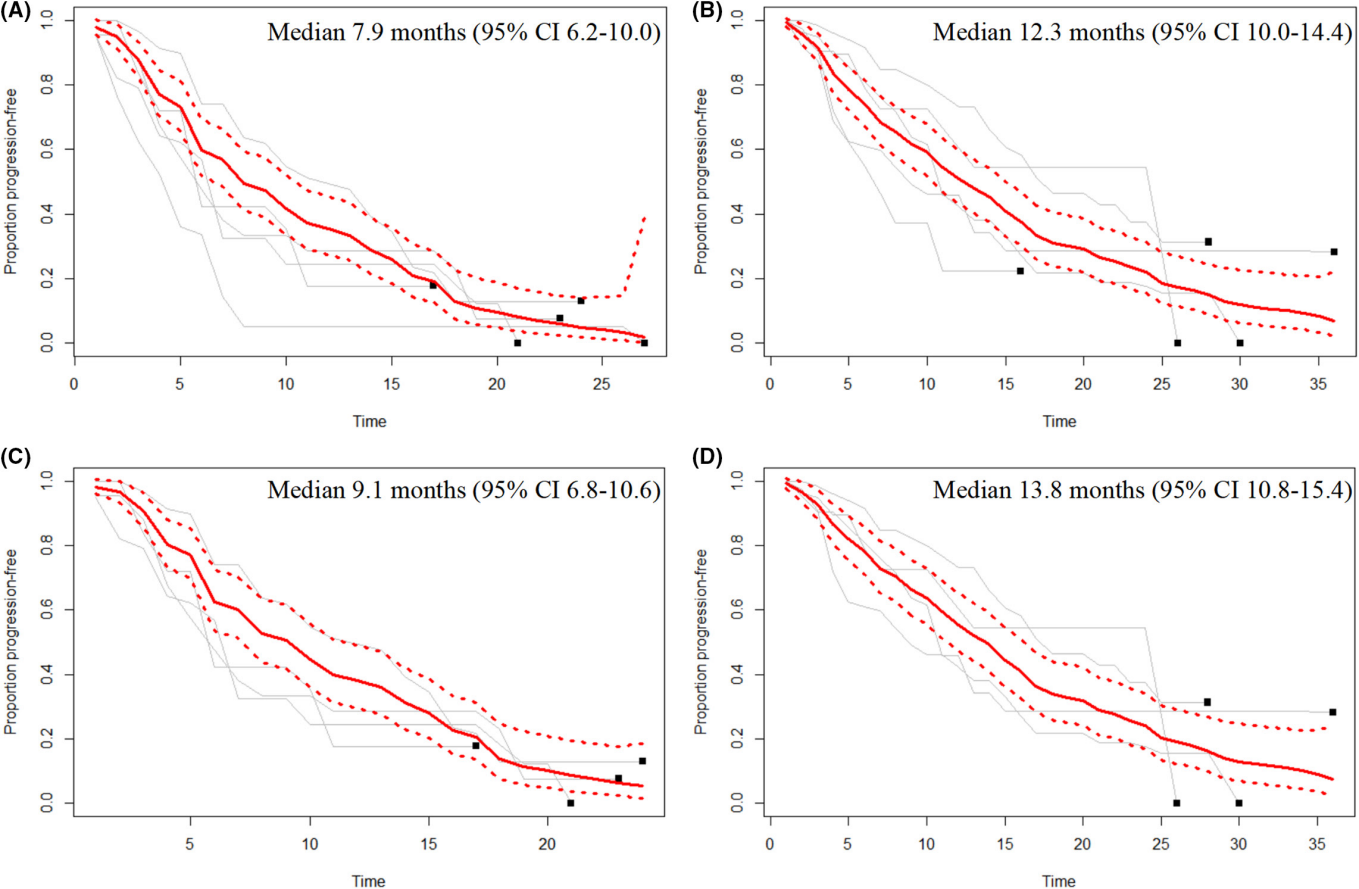
Curves of the summary progression‐free survival (PFS) and overall survival (OS). (A) TKI‐containing regimens for PFS in the five studies of the meta‐analysis; (B) TKI‐containing regimens for OS in the five studies of the meta‐analysis; (C) TKI + Cap‐containing regimens for PFS in the four studies of the meta‐analysis; (D) TKI + Cap‐containing regimens for OS in the four studies of the meta‐analysis. Notes: The gray lines represent survival in each study, and the black square represents the end of follow‐up. The thick lines represent summarized survival curves with 95% confidence intervals (dashed lines) obtained using MetaSurv with a fixed‐effects model.

Five arms from four trials reported the PFS K‐M curves, and the summary PFS and 95% CI curves are shown in Figure 4A with the fixed‐effects model. We also found favorable efficacy of TKI‐containing regimens and TKI + Cap‐containing regimen subgroups, based on the analysis of survival to an outcome event. The summarized PFS curves of patients treated with TKIs have showed the summary median PFS time was 7.9 months (95% CI: 6.2–10.0; *I* ^2^ = 0.00%). While TKI + Cap‐containing regimens subgroup showed an obviously higher summary median PFS time of 9.1 months (95% CI: 6.8–10.6; *I* ^2^ = 0.00%) (Figure 4C). The OS K‐M curves were presented in five trials; the summary median OS time using the fixed‐effects model was 12.3 months (95% CI: 10.0–14.4; *I* ^2^ = 0.00%) with TKI‐containing regimens treatment (Figure 4B). While four studies presented the OS K‐M curves, the summary median OS time was 13.8 months (95% CI: 10.8–15.4; *I* ^2^ = 0.00%) in TKI + Cap‐containing regimens subgroup (Figure 4D).

However, Lapatinib‐containing regimens subgroup reported a slightly lower summary median PFS time with 5.90 months (95% CI: 3.92–6.97; *I* ^2^ = 0.00%) (Figure S3) compared with TKI‐containing regimens. However, only HER2CLIMB reported the median PFS time of BCBM with Tucatinib‐containing treatment (9.9 months; 95% CI: 8.0–13.9); PHENIX presented the median PFS time of pyrotinib‐containing regimen for people with BCBM (6.9 months; 95% CI: 5.4‐not reached); also TBCRC 022 reported the median PFS time of BCBM with the treatment of neratinib‐containing regimen (1.9 months) (Table 1). As Lapatinib‐containing regimens treatment, four trials reported the OS K‐M curves; the summary median OS time was 12.99 months (95% CI: 10.30–14.92) (Figure S3). However, only HER2CLIMB reported the median OS time with tucatinib‐containing regimen treatment (18.1 months; 15.5‐not reached); only TBCRC 022 investigating neratinib‐containing treatment presented the median OS time (8.7 months) (Table 1). In conclusion, tucatinib‐containing regimens appear to be the most favorable TKIs for BCBM patients, with better prognosis.

**TABLE 1:** Comparison of median progression‐free survival (PFS) and overall survival (OS) time in different types of TKI for HER2‐positive BCBM patients.

TKIs	Median PFS time	Median OS time
Lapatinib^a^	5.90 months (95% CI: 3.92–6.97)	12.99 months (95% CI: 10.30–14.92)
Tucatinib^16^	9.9 months (95% CI: 8.0–13.9)	18.1 months (95% CI: 15.5‐not reached)
Neratinib^27^, ^28^	1.9 months	8.7 months
Pyrotinib^31^	6.9 months (95% CI: 5.4‐not reached)	NA

Abbreviations: OS, overall survival; NA: not available; PFS: progression‐free survival; TKI: Tyrosine kinase inhibitors.

^a^ The results were calculated as the area under the summary survival curve using MetaSurv with a fixed‐effects model.

### 3.4 Toxicity

**FIGURE 5:**
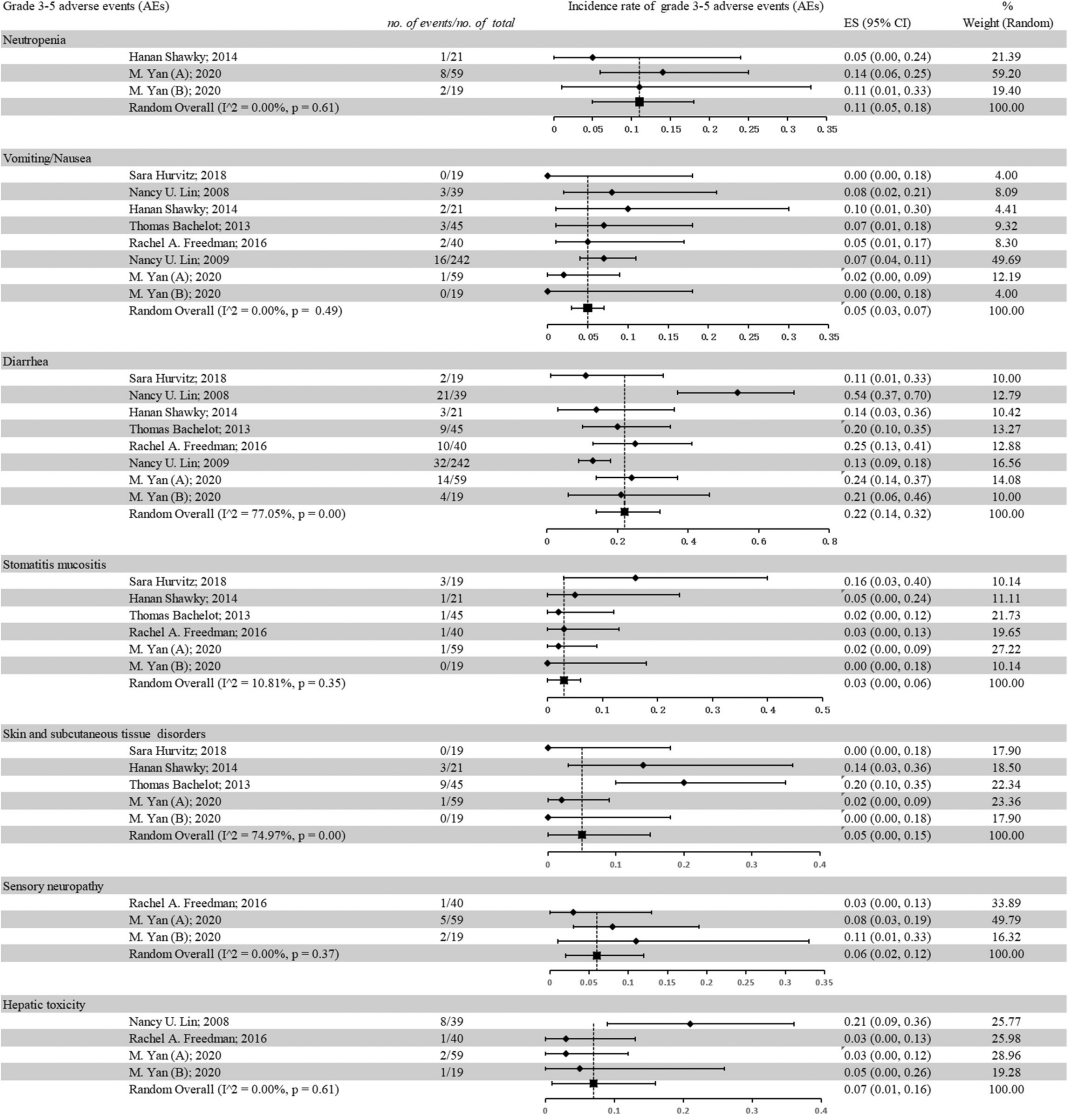
Pooled grade 3–5 adverse events in HER2‐positive breast cancer brain metastases patients included.

We generated grade 3–5 AEs that occurred most frequently during treatment, including neutropenia, vomiting/nausea, diarrhea, stomatitis, mucositis, skin and subcutaneous tissue disorders, sensory neuropathy, and hepatic toxicity. In general, the pooled analysis results showed that the safety outcomes of the TKI‐containing regimens were acceptable. The common toxicities with grade 3–5 AEs during treatment with TKI‐containing regimens included diarrhea (22%, 95% CI: 14%–32%), neutropenia (11%, 95% CI: 5%–18%), hepatic toxicity (7%, 95% CI: 1%–16%), and sensory neuropathy (6%, 95% CI: 2%–12%). Compiled grade 3–5 AEs in HER2‐positive BCBM patients are shown in Figure 5.

## 4 DISCUSSION

Survival outcomes of patients with HER2‐positive BC have greatly improved with the availability of additional treatment approaches. However, an increase in the incidence of CBM has been reported over the last few years. This may be due to the combination of poor penetrability of the BBB to chemotherapy drugs, monoclonal antibodies, and antibody‐dependent cell‐mediated cytotoxicity (ADCC) drugs, allowing the CNS to become a “shelter” for cancer cells, and advanced medical imaging technology becoming better at detecting brain metastatic lesions.^32^, ^33^

The presupposition for disease control and overall efficacy in treating CBM is adequate target engagement and drug penetration into the brain and tumors.^34^ Among the trials included in this meta‐analysis, trastuzumab was included in most previous lines of therapy, and the majority of patients had previously received brain radiation therapy, including WBRT or SRS. Our meta‐analysis showed that TKI‐containing regimens are an effective treatment protocol for HER2‐positive BCBM patients, with favorable summary median PFS and OS (7.9 months, 12.3 months; respectively). Small‐molecule TKIs have a lower molecular weight, which allows them to effectively penetrate the BBB.^35^ In addition, TKI drugs inhibit both constitutive and ligand‐induced ErbB signaling compared to monoclonal antibodies.^35^ The unique physicochemical properties mentioned above have produced advantageous results. The efficacy of TKIs has been confirmed in animal models of preclinical HER2‐positive BCBM animal models. Gril et al. found that animal models treated with lapatinib had fewer large metastatic sites than those of patients treated with vehicle.^36^ Furthermore, clinical trials have revealed the efficacy and toxicity of TKI‐containing regimens in HER2‐positive BCBM patients.^16^, ^17^, ^22^, ^37^

Subgroup analysis showed that the TKI + Cap regimens had a more substantial benefit with a favorable CNS ORR of 43% compared with that of TKI (monotherapy) (5%) and TKI + Others (11%) and produced longer median PFS and OS. These results are consistent with those of a phase II trial that assessed lapatinib efficacy and reported a CNS ORR of 6%. Of the 50 patients who entered the capecitabine extension phase, 20% achieved an objective CNS response.^29^ Chefrour et al. attempted to elucidate the reason for the improved efficacy of this combination through molecular determinants of the response. They indicated that lapatinib and capecitabine could regulate each other's molecular determinants of response, and this synergistic interaction was also observed in subcutaneous BC mouse models.^38^ All of these results suggest that of all TKI‐containing strategies (TKI Monotherapy or combined with other drugs), the combination of TKI and capecitabine is the most effective treatment method for HER2‐positive BCBM patients.

The TKIs available for clinical practice or under investigation mainly include lapatinib, neratinib, afatinib, pyrotinib, and tucatinib. However, the optimal treatment for HER2‐positive BCBM remains controversial. We performed a subgroup analysis to explore the efficacy of the different TKIs. Owing to limited data, we could not perform any summary analysis apart from Lapatinib‐containing regimens. However, the HER2CLIMB showed significantly better outcomes of a tucatinib‐containing regimen of 9.9 months for PFS and 18.1 months for OS compared with those of lapatinib‐containing regimens of 5.90 months for PFS and 12.99 months for OS.^39^ Jing et al. studied the advantages of tucatinib using mechanistic modeling of CNS pharmacokinetics and target engagement of TKIs, including lapatinib, neratinib, and tucatinib. As revealed by the physiologically based pharmacokinetic model approach, tucatinib can induce sufficient HER2 inhibition in both disrupted and largely intact BBB; conversely, lapatinib and neratinib may not achieve adequate target inhibition.^34^ These mechanistic models are in line with the available efficacy data, and our results support the pharmacological value of tucatinib for the treatment of HER2‐positive BCBM patients. Therefore, based on the current data, tucatinib may be the most effective TKI.

HER2‐targeting antibody therapies, including trastuzumab, trastuzumab emtansine (T‐DM1), trastuzumab deruxtecan (T‐DXd), and pertuzumab, were discovered in a medical history. Generally, they have limited roles in treating BM as first‐line therapy because of the BBB. However, recent studies have suggested the opposite view, as some clinical trials have shown that large‐molecule drugs can be effective treatments for BCBM.^40^, ^41^, ^42^, ^43^, ^44^ The results of preclinical trials may explain these findings. The BBB can be disturbed during brain metastasis and replaced by the blood‐tumor barrier, which has better drug permeability.^45^, ^46^ Dijkers et al. found that ^89^Zr‐trastuzumab was detected in brain lesions at an 18‐fold higher concentration than that in normal brain tissue, which supports the feasibility of HER2‐targeting therapy in patients with BCBM.^47^ Clinical trial results also support this phenomenon, showing a CNS response to ADCC drugs. KAMILLA, a single‐arm phase IIIb trial, evaluated the efficacy of T‐DM1 in patients with metastatic BC, and a subgroup analysis demonstrated that T‐DM1 extended PFS and OS in patients with baseline BM.^42^ T‐DM1 is no longer the newest HER2‐targeted ADCC drug, T‐DXd. T‐DXd also appeared to affect BCBM. In the randomized DESTINY‐Breast03 study, T‐DXd was found to significantly increase PFS in patients with BM.^43^, ^44^ However, these data were compared in patients with stable brain metastases at baseline. Therefore, a single‐arm phase II trial called TUXEDO‐1 enrolled patients with HER2‐positive BC with active BM, and the results showed that T‐DXd still had a remarkable CNS ORR (73.3%).^43^ However, direct head‐to‐head comparisons of TKI and ADCC drugs are lacking. Currently, TKIs play a leading role in the treatment of BCBM patients.

To our knowledge, this study is the first to assess the efficacy and safety of TKI‐containing regimens for the treatment of HER2‐positive BCBM. Our meta‐analysis included five RCTs and eight single‐arm clinical trials of high quality and integrity. A pairwise meta‐analysis was performed to compare the efficacy of TKI‐containing regimens and non‐TKI regimens. Pooled analyses and summary survival curves obtained from single‐arm studies provided quantitative and intuitive results. However, trial design discrepancies between different studies can account for the high heterogeneity of pooled analyses in single‐arm studies. Subgroup analysis was used to reduce heterogeneity and yielded reliable results.

## 5 CONCLUSION

TKI therapy improves the survival outcomes of HER2‐positive BCBM patients, especially when combined with capecitabine and tolerable AEs. We also paid attention to the clinical value of tucatinib, which appears to be the most favorable TKI drug for BCBM patients.

## AUTHOR CONTRIBUTIONS

**Yushuai Yu:** Conceptualization (equal); data curation (equal); formal analysis (equal); methodology (equal); writing – original draft (equal). **Kaiyan Huang:** Formal analysis (equal); methodology (equal); writing – original draft (equal). **Yuxiang Lin:** Methodology (equal); writing – review and editing (equal). **Jie Zhang:** Data curation (equal); methodology (equal); writing – original draft (equal). **Chuangui Song:** Conceptualization (equal); writing – review and editing (equal).

## FUNDING INFORMATION

This study was not supported by any financial or personal relationships with other people or organizations.

## CONFLICT OF INTEREST STATEMENT

The authors have declared that no competing interests exist.

## PATIENT CONSENT

This article does not include studies with human participants conducted by any author.

## INFORMED CONSENT

As this study contained data released from the published literature, informed consent was not required.

## Supporting information


Table S1

Appendix S1

Appendix S2

Figure S1

Figure S2

Figure S3

## Data Availability

All data generated or analyzed in this study are included in the published article [and its supplementary information files].

## References

[1] Siegel RL , Miller KD , Fuchs HE , Jemal A . Cancer statistics, 2022. CA Cancer J Clin. 2022;72(1):7‐33. 35020204 10.3322/caac.21708

[2] Fecci PE , Champion CD , Hoj J , et al. The evolving modern Management of Brain Metastasis. Clin Cancer Res. 2019;25(22):6570‐6580. 31213459 10.1158/1078-0432.CCR-18-1624 PMC8258430

[3] Waks AG , Winer EP . Breast cancer treatment: a review. JAMA. 2019;321(3):288‐300. 30667505 10.1001/jama.2018.19323

[4] Martin AM , Cagney DN , Catalano PJ , et al. Brain metastases in newly diagnosed breast cancer: a population‐based study. JAMA Oncol. 2017;3(8):1069‐1077. 28301662 10.1001/jamaoncol.2017.0001 PMC5824221

[5] Kuksis M , Gao Y , Tran W , et al. The incidence of brain metastases among patients with metastatic breast cancer: a systematic review and meta‐analysis. Neuro‐Oncology. 2021;23(6):894‐904. 33367836 10.1093/neuonc/noaa285 PMC8168821

[6] Brufsky AM , Mayer M , Rugo HS , et al. Central nervous system metastases in patients with HER2‐positive metastatic breast cancer: incidence, treatment, and survival in patients from registHER. Clin Cancer Res. 2011;17(14):4834‐4843. 21768129 10.1158/1078-0432.CCR-10-2962

[7] Patchell RA , Tibbs PA , Regine WF , et al. Postoperative radiotherapy in the treatment of single metastases to the brain: a randomized trial. JAMA. 1998;280(17):1485‐1489. 9809728 10.1001/jama.280.17.1485

[8] Mekhail T , Sombeck M , Sollaccio R . Adjuvant whole‐brain radiotherapy versus observation after radiosurgery or surgical resection of one to three cerebral metastases: results of the EORTC 22952‐26001 study. Curr Oncol Rep. 2011;13(4):255‐258. 21584645 10.1007/s11912-011-0180-1

[9] Mahajan A , Ahmed S , McAleer MF , et al. Post‐operative stereotactic radiosurgery versus observation for completely resected brain metastases: a single‐Centre, randomised, controlled, phase 3 trial. Lancet Oncol. 2017;18(8):1040‐1048. 28687375 10.1016/S1470-2045(17)30414-X PMC5560102

[10] Soffietti R , Abacioglu U , Baumert B , et al. Diagnosis and treatment of brain metastases from solid tumors: guidelines from the European Association of Neuro‐Oncology (EANO). Neuro‐Oncology. 2017;19(2):162‐174. 28391295 10.1093/neuonc/now241 PMC5620494

[11] Lippitz B , Lindquist C , Paddick I , Peterson D , O'Neill K , Beaney R . Stereotactic radiosurgery in the treatment of brain metastases: the current evidence. Cancer Treat Rev. 2014;40(1):48‐59. 23810288 10.1016/j.ctrv.2013.05.002

[12] Raman S , Mou B , Hsu F , et al. Whole Brain radiotherapy versus stereotactic radiosurgery in poor‐prognosis patients with one to 10 Brain metastases: a randomised feasibility study. Clin Oncol (R Coll Radiol). 2020;32(7):442‐451. 32085923 10.1016/j.clon.2020.02.001

[13] Rusnak DW , Affleck K , Cockerill SG , et al. The characterization of novel, dual ErbB‐2/EGFR, tyrosine kinase inhibitors: potential therapy for cancer. Cancer Res. 2001;61(19):7196‐7203. 11585755

[14] Moulder SL , Borges VF , Baetz T , et al. Phase I study of ONT‐380, a HER2 inhibitor, in patients with HER2(+)‐advanced solid tumors, with an expansion cohort in HER2(+) metastatic breast cancer (MBC). Clin Cancer Res. 2017;23(14):3529‐3536. 28053022 10.1158/1078-0432.CCR-16-1496

[15] Bachelot T , Romieu G , Campone M , et al. Lapatinib plus capecitabine in patients with previously untreated brain metastases from HER2‐positive metastatic breast cancer (LANDSCAPE): a single‐group phase 2 study. Lancet Oncol. 2013;14(1):64‐71. 23122784 10.1016/S1470-2045(12)70432-1

[16] Lin NU , Murthy RK , Abramson V , et al. Tucatinib vs placebo, both in combination with trastuzumab and capecitabine, for previously treated ERBB2 (HER2)‐positive metastatic breast cancer in patients with Brain metastases: updated exploratory analysis of the HER2CLIMB randomized clinical trial. JAMA Oncol. 2022;9:197‐205. 10.1001/jamaoncol.2022.5610 PMC9716438 36454580

[17] Yan M , Ouyang Q , Sun T , et al. Pyrotinib plus capecitabine for patients with human epidermal growth factor receptor 2‐positive breast cancer and brain metastases (PERMEATE): a multicentre, single‐arm, two‐cohort, phase 2 trial. Lancet Oncol. 2022;23(3):353‐361. 35085506 10.1016/S1470-2045(21)00716-6

[18] Freedman RA , Gelman RS , Wefel JS , et al. Translational breast cancer research consortium (TBCRC) 022: a phase II trial of neratinib for patients with human epidermal growth factor receptor 2‐positive breast cancer and Brain metastases. J Clin Oncol. 2016;34(9):945‐952. 26834058 10.1200/JCO.2015.63.0343 PMC5070554

[19] Combescure C , Foucher Y , Jackson D . Meta‐analysis of single‐arm survival studies: a distribution‐free approach for estimating summary survival curves with random effects. Stat Med. 2014;33(15):2521‐2537. 24532219 10.1002/sim.6111

[20] Slim K , Nini E , Forestier D , Kwiatkowski F , Panis Y , Chipponi J . Methodological index for non‐randomized studies (minors): development and validation of a new instrument. ANZ J Surg. 2003;73(9):712‐716. 12956787 10.1046/j.1445-2197.2003.02748.x

[21] Krop IE , Lin NU , Blackwell K , et al. Trastuzumab emtansine (T‐DM1) versus lapatinib plus capecitabine in patients with HER2‐positive metastatic breast cancer and central nervous system metastases: a retrospective, exploratory analysis in EMILIA. Annals of Oncology. 2015;26(1):113‐119. 25355722 10.1093/annonc/mdu486 PMC4679405

[22] Takano T , Tsurutani J , Takahashi M , et al. A randomized phase II trial of trastuzumab plus capecitabine versus lapatinib plus capecitabine in patients with HER2‐positive metastatic breast cancer previously treated with trastuzumab and taxanes: WJOG6110B/ELTOP. Breast. 2018;40:67‐75. 29698927 10.1016/j.breast.2018.04.010

[23] Cortés J , Dieras V , Ro J , et al. Afatinib alone or afatinib plus vinorelbine versus investigator's choice of treatment for HER2‐positive breast cancer with progressive brain metastases after trastuzumab, lapatinib, or both (LUX‐breast 3): a randomised, open‐label, multicentre, phase 2 trial. Lancet Oncol. 2015;16(16):1700‐1710. 26596672 10.1016/S1470-2045(15)00373-3

[24] Hurvitz S , Singh R , Adams B , et al. Phase Ib/II single‐arm trial evaluating the combination of everolimus, lapatinib and capecitabine for the treatment of HER2‐positive breast cancer with brain metastases (TRIO‐US B‐09). Ther Adv Med Oncol. 2018;10:1758835918807339. 30542377 10.1177/1758835918807339 PMC6236634

[25] Lin NU , Carey LA , Liu MC , et al. Phase II trial of lapatinib for brain metastases in patients with human epidermal growth factor receptor 2‐positive breast cancer. J Clin Oncol. 2008;26(12):1993‐1999. 18421051 10.1200/JCO.2007.12.3588 PMC4524351

[26] Shawky H , Tawfik H . All‐oral combination of lapatinib and capecitabine in patients with brain metastases from HER2‐positive breast cancer – a phase II study. J Egypt Natl Canc Inst. 2014;26(4):187‐194. 25294797 10.1016/j.jnci.2014.08.001

[27] Freedman RA , Gelman RS , Wefel JS , et al. Translational breast cancer research consortium (TBCRC) 022: a phase II trial of neratinib for patients with human epidermal growth factor receptor 2‐positive breast cancer and Brain metastases. Journal of clinical oncology official journal of the American society of. Clin Oncol. 2016;34:945‐952. 10.1200/JCO.2015.63.0343 PMC5070554 26834058

[28] Freedman RA , Gelman RS , Melisko ME , Anders CK , Lin NU . TBCRC 022: phase II trial of neratinib + capecitabine for patients (pts) with human epidermal growth factor receptor 2 (HER2+) breast cancer brain metastases (BCBM). J Clin Oncol. 2017;35(15_suppl):1005.

[29] Lin NU , Diéras V , Paul D , et al. Multicenter phase II study of lapatinib in patients with brain metastases from HER2‐positive breast cancer. Clin Cancer Res. 2009;15(4):1452‐1459. 19228746 10.1158/1078-0432.CCR-08-1080

[30] Leone JP , Duda DG , Hu J , et al. A phase II study of cabozantinib alone or in combination with trastuzumab in breast cancer patients with brain metastases. Breast Cancer Res Treat. 2020;179(1):113‐123. 31541381 10.1007/s10549-019-05445-z

[31] Yan M , Bian L , Hu X , et al. Pyrotinib plus capecitabine for human epidermal factor receptor 2‐positive metastatic breast cancer after trastuzumab and taxanes (PHENIX): a randomized, double‐blind, placebo‐controlled phase 3 study. Transl Breast Cancer Res. 2020;1:1.

[32] Gobbini E , Ezzalfani M , Dieras V , et al. Time trends of overall survival among metastatic breast cancer patients in the real‐life ESME cohort. Eur J Cancer. 2018;96:17‐24. 29660596 10.1016/j.ejca.2018.03.015

[33] Bailleux C , Eberst L , Bachelot T . Treatment strategies for breast cancer brain metastases. Br J Cancer. 2021;124(1):142‐155. 33250512 10.1038/s41416-020-01175-y PMC7782834

[34] Li J , Jiang J , Bao X , et al. Mechanistic modeling of central nervous system pharmacokinetics and target engagement of HER2 tyrosine kinase inhibitors to inform treatment of breast cancer Brain metastases. Clin Cancer Res. 2022;28(15):3329‐3341. 35727144 10.1158/1078-0432.CCR-22-0405 PMC9357092

[35] Duchnowska R , Loibl S , Jassem J . Tyrosine kinase inhibitors for brain metastases in HER2‐positive breast cancer. Cancer Treat Rev. 2018;67:71‐77. 29772459 10.1016/j.ctrv.2018.05.004

[36] Gril B , Palmieri D , Bronder JL , et al. Effect of lapatinib on the outgrowth of metastatic breast cancer cells to the brain. J Natl Cancer Inst. 2008;100(15):1092‐1103. 18664652 10.1093/jnci/djn216 PMC2575427

[37] Seligmann JF , Wright‐Hughes A , Pottinger A , et al. Lapatinib plus capecitabine versus trastuzumab plus capecitabine in the treatment of human epidermal growth factor receptor 2‐positive metastatic breast cancer with central nervous system metastases for patients currently or previously treated with trastuzumab (LANTERN): a phase II randomised trial. Clin Oncol (R Coll Radiol). 2020;32(10):656‐664. 32600919 10.1016/j.clon.2020.06.003

[38] Chefrour M , Milano G , Formento P , et al. Positive interaction between lapatinib and capecitabine in human breast cancer models: study of molecular determinants. Fundam Clin Pharmacol. 2012;26(4):530‐537. 21623901 10.1111/j.1472-8206.2011.00945.x

[39] Lin NU , Borges V , Anders C , et al. Intracranial efficacy and survival with Tucatinib plus trastuzumab and capecitabine for previously treated HER2‐positive breast cancer with Brain metastases in the HER2CLIMB trial. J Clin Oncol. 2020;38(23):2610‐2619. 32468955 10.1200/JCO.20.00775 PMC7403000

[40] Park YH , Park MJ , Ji SH , et al. Trastuzumab treatment improves brain metastasis outcomes through control and durable prolongation of systemic extracranial disease in HER2‐overexpressing breast cancer patients. Br J Cancer. 2009;100(6):894‐900. 19240719 10.1038/sj.bjc.6604941 PMC2661774

[41] Swain SM , Baselga J , Miles D , et al. Incidence of central nervous system metastases in patients with HER2‐positive metastatic breast cancer treated with pertuzumab, trastuzumab, and docetaxel: results from the randomized phase III study CLEOPATRA. Ann Oncology. 2014;25(6):1116‐1121. 10.1093/annonc/mdu133 PMC4037862 24685829

[42] Montemurro F , Delaloge S , Barrios CH , et al. Trastuzumab emtansine (T‐DM1) in patients with HER2‐positive metastatic breast cancer and brain metastases: exploratory final analysis of cohort 1 from KAMILLA, a single‐arm phase IIIb clinical trial(☆). Ann Oncology. 2020;31(10):1350‐1358. 10.1016/j.annonc.2020.06.020 32634611

[43] Bartsch R , Berghoff AS , Furtner J , et al. Trastuzumab deruxtecan in HER2‐positive breast cancer with brain metastases: a single‐arm, phase 2 trial. Nat Med. 2022;28(9):1840‐1847. 35941372 10.1038/s41591-022-01935-8 PMC9499862

[44] Cortés J , Kim SB , Chung WP , et al. Trastuzumab Deruxtecan versus Trastuzumab Emtansine for Breast Cancer. N Engl J Med. 2022;386(12):1143‐1154. 35320644 10.1056/NEJMoa2115022

[45] Arvanitis CD , Ferraro GB , Jain RK . The blood‐brain barrier and blood‐tumour barrier in brain tumours and metastases. Nat Rev Cancer. 2020;20(1):26‐41. 31601988 10.1038/s41568-019-0205-x PMC8246629

[46] Eichler AF , Chung E , Kodack DP , Loeffler JS , Fukumura D , Jain RK . The biology of brain metastases‐translation to new therapies. Nat Rev Clin Oncol. 2011;8(6):344‐356. 21487419 10.1038/nrclinonc.2011.58 PMC3259742

[47] Dijkers EC , Oude Munnink TH , Kosterink JG , et al. Biodistribution of 89Zr‐trastuzumab and PET imaging of HER2‐positive lesions in patients with metastatic breast cancer. Clin Pharmacol Ther. 2010;87(5):586‐592. 20357763 10.1038/clpt.2010.12

