# Supplementary material for: Tyrosine kinase inhibitors in HER2‐positive breast cancer brain metastases: A systematic review and meta‐analysis
Source: Cancer Med. 2023 May 31;12(14):15090–100. doi: 10.1002/cam4.6180 (PMC10417165; doi:10.1002/cam4.6180)
Supplement: Supplementary file 1 — Table S1 [file CAM4-12-15090-s003.doc]

**Table S1. Characteristics of eligible studies**

| **Randomized controlled trials (RCTs)** | | | | | | | | | | | | | |
| --- | --- | --- | --- | --- | --- | --- | --- | --- | --- | --- | --- | --- | --- |
| **NO.** | **Author;**  **Year** | **Study name**  **[Clinicaltrial.gov ID]** | | **Locations** | | **Phase** | **Treatment regimens** | **Treatment**  **details** | **ECOG PS** | **Of Analyzed**  **Participants** | **CNS ORR** | **PFS**  **(months)** | **OS**  **(months)** |
| 1 | I. E. Krop,2014(21) | EMILIA | | North America, South America, Europe, Asia, Oceania | | phase III | T-DM1 | T-DM1 3.6 mg/kg i.v. every 21 days | 0-1 | 45 | NA | 5.9; HR=1.00 (0.54–1.84) | 26.8; HR=0.38(0.184−0.795) |
|  |  |  |  |  |  |  | LX | X 1000 mg/m2 orally twice-daily on days 1–14 of each 21-day cycle, and L 1250 mg orally once-daily on days 1–21. | 0-1 | 50 | NA | 5.7 | 12.9 |
| 2 | Toshimi Takano,2018 (22) | WJOG6110B (ELTOP) | | Asia | | phase II | LX | L 1250 mg/day, and X 2000 mg/  m2/day on days 1-14 every 3 weeks. | 0-2 | 7 | NA | HR=0.62 (0.18–2.17) | NA |
|  |  |  |  |  |  |  | HX | H 4 mg/kg loading then 2 mg/kg weekly or 8 mg/kg loading then 6 mg/kg every 3 weeks, and X 2500 mg/m2/day on days 1-14 every 3 weeks. | 0-2 | 6 | NA | NA | NA |
| 3 | Nancy U.Lin,2020 (16) | HER2CLIMB | | North America, Oceania, Europe, | | phase II | T+HX | T 300 mg orally twice daily, and H 8 mg/kg i.v. on Day 1 of Cycle 1, followed by 6 mg/kg on Day1 of each 21-day cycle, and X 1000 mg/m2 orally twice daily on Days 1-14 of each 21-day cycle. | 0-1 | 198 | NA | 9.9 (8.0- 13.9); HR=0.32 (0.22-0.48) | 18.1(15.5 to –); HR=0.58(0.40-0.85) |
|  |  |  |  |  |  |  | HX | H 8 mg/kg i.v. on Day 1 of Cycle 1, followed by 6 mg/kg on Day 1 of each 21-day cycle, and X 1000 mg/m2 orally twice daily on Days 1-14 of each 21-day cycle. | 0-1 | 93 | NA | 4.2  (3.6-5.7) | 12.0 (12.2-15.2) |
| 4 | Min Yan, 2020 (31) | PHENIX | | Asia | | phase III | PX | P 400 mg once daily and X 1000 mg/m2 per day on day 1 through 14, every 21 days. | 0-1 | 21 | NA | 6.9  (5.4to-);  HR=0.32  (0.13-0.77) | NA |
|  |  |  |  |  |  |  | X | Placebo 400 mg once daily, and X  1000 mg/m2 per day on day 1 through 14, every 21 days. | 0-1 | 10 | NA | 4.2 (0.8-6.9) | NA |
| 5 | Nadia Harbeck,2016 (23) | LUX-Breast 1 | North America, South America, Europe, Asia, Africa | | | phase III | AV | A (40 mg/day) once daily, and intravenous V (25 mg/m² per week). | 0-1 | 43 | NA | HR=1.318 (0.670-2.593) | HR=1.551 (0.728-3.307) |
|  |  |  |  |  |  |  | HV | Intravenous H (2 mg/kg  per week after 4 mg/kg loading dose), and intravenous V (25 mg/m² per week). | 0-2 | 17 |  |  |  |
| **Single-arm clinical trials** | | | | | | | | | | | | | |
| **NO.** | **Author;**  **Year** | **Study name**  **[Clinicaltrial.gov ID]** | | | **Locations** | **Phase** | **Treatment regimens** | **Treatment**  **details** | **ECOG PS** | **Of Analyzed**  **Participants** | **CNS ORR** | **PFS**  **(months)** | **OS**  **(months)** |
| 1 | Sara Hurvitz, 2018 (24) | TRIO-US B-09 | | | North America | Phase Ib/II | LXE | L po qd and E po qd on days 1-21, and X po bid on days 1-14 (Treatment repeats every 21 days for 17 courses in the absence of disease progression or unacceptable toxicity). | 0-2 | 11 | 27%  (3.6–41.4) | NA | NA |
| 2 | Nancy U. Lin, 2008 (25) |  | | | North America | Phase II | L | L was 750 mg twice daily administered orally in continuous 4-week cycles. | 0-2 | 39 | 2.6%  (0.21-26) | NA | 6.6 (4.4-11)* |
| 3 | Hanan Shawky, 2014 (26) |  | | | Africa | Phase II | LX | L 1250 mg once daily every morning  continuously and X 2000 mg/m2/day, divided into two doses, on days 1–14, every 21 days. | 0-2 | 21 | 33.3% | 5.5 (1.1–22.0) | 11 |
| 4 | Thomas Bachelot, 2013 (15) | LANDSCAPE(NCT00967031) | | Europe | | Phase II | LX | X 2000 mg/m² every day from day 1 to day 14 every 21 days, and L at the approved dose of 1250 mg every  day continuously. | 0-2 | 45 | 65.9% (50.1–79.5) | NA | 17.0 (13.7–24.9) |
| 5 | Rachel A. Freedman, 2016 (27, 28) | TBCRC 022 | | | North America | Phase II | N | N 240 mg was administered once per day without breaks, and cycle duration was 28 days. | 0-2 | 40 | 8%  (2-22) | 1.9 | 8.7 |
| 6 | Nancy U. Lin, 2009 (29) | NCT00263588 | | | North America, Europe, Asia, Oceania | Phase II | L | L 750 mg bid (Dose delays of up to 2 weeks and two dose reductions, first to 1,500 mg qd and second  to 1,250 mg qd, were allowed for treatment-related toxicities). | 0-3 | 242 | 6%  (3.6-10.2) | 2.4 (1.87-2.79) | 6.37 (5.49-8.25) |
| 7 | M. Yan, 2020^†^ (17) | PERMEATE (NCT03691051) | | | Asia | Phase II | PX (cohort A) | P 400mg qd and X 1000 mg/m2 bid for 14 days and no drug for 7 days. | 0-2 | 59 | 74.6% (  61.6-85.0) | 11.3 (7.7-14.6) | NA |
|  |  |  |  |  |  |  | PX (cohort B) | P 400mg qd and X 1000 mg/m2 bid for 14 days and no drug for 7 days. | 0-2 | 19 | 42.1% (20.3-66.5) | 5.6 (3.4-10.0) | NA |
| 8 | José Pablo Leone, 2019 (30) | NCT02260531 | | | North America | Phase II | HC | C 60 mg qd, during a 21-day cycle and H 8 mg/kg IV loading dose followed by 6 mg/kg IV every 3 weeks. | 0-1 | 21 | 5% (0.2-23) | 4.1 (2.8-6.2) | 13.8 (8.2, NA) |
| **Abbreviations:**  1. ECOG PS: Eastern Cooperative Oncology Group Performance Status; CNS ORR: central nervous system objective response rate; CNS TTP: central nervous system time to progress; PFS: progression-free survival; OS: overall survival;  2. Drugs: L: lapatinib; X: capecitabine; H: trastuzumab; T: tucatinib; P: Pyrotinib; A: Afatinib; V: vinorelbine; T: topotecan; E: everolimus; N: neratinib; C: cabozantinib  3.NA: not available  ^†^PERMEATE was divided into two cohorts whether patients with previous radiotherapy.  ‡Data were record by GetData Graph Digitizer if the original statistical curve picture was acquired from articles. | | | | | | | | | | | | | |
