# Supplementary material for: Tyrosine kinase inhibitors in HER2‐positive breast cancer brain metastases: A systematic review and meta‐analysis
Source: Cancer Med. 2023 May 31;12(14):15090–100. doi: 10.1002/cam4.6180 (PMC10417165; doi:10.1002/cam4.6180)
Supplement: Supplementary file 3 — Appendix S2 [file CAM4-12-15090-s006.doc]

**Appendix S2.** Quality assessment of included trials

1. Bias assessment of randomized controlled trials (RCTs).

**
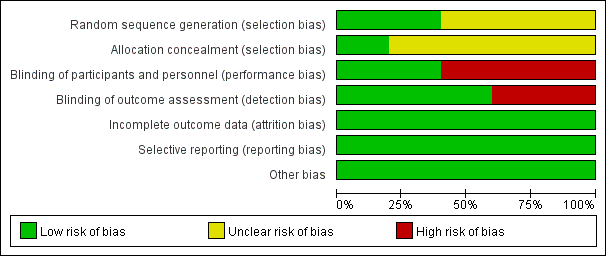
**

1. Quality assessment of single-arm clinical trials.

| **MINORS index for included non-randomized studies.** | | | | | | | | | |
| --- | --- | --- | --- | --- | --- | --- | --- | --- | --- |
| Study | Ⅰ | Ⅱ | Ⅲ | Ⅳ | Ⅴ | Ⅵ | Ⅶ | Ⅷ | Total |
| Sara Hurvitz, 2018 | 2 | 2 | 2 | 2 | 0 | 2 | 2 | 2 | 14 |
| Nancy U. Lin, 2008 | 2 | 2 | 2 | 2 | 0 | 2 | 2 | 2 | 14 |
| Hanan Shawky, 2014 | 2 | 2 | 2 | 2 | 0 | 2 | 2 | 2 | 14 |
| Thomas Bachelot, 2013 | 2 | 2 | 2 | 2 | 0 | 2 | 2 | 2 | 14 |
| Rachel A. Freedman, 2016 | 2 | 2 | 2 | 2 | 0 | 2 | 2 | 2 | 14 |
| Nancy U. Lin, 2009 | 2 | 2 | 2 | 2 | 1 | 2 | 2 | 2 | 15 |
| M. Yan, 2022 | 2 | 2 | 2 | 2 | 0 | 2 | 2 | 2 | 14 |
| José Pablo Leone, 2019 | 2 | 2 | 2 | 2 | 0 | 2 | 2 | 2 | 14 |

**Note:** Numbers I-Ⅷ in heading signified: Ⅰ, a clearly stated aim; Ⅱ, inclusion of consecutive patients; Ⅲ, prospective collection of data; Ⅳ, endpoints appropriate to the aim of the study; Ⅴ, unbiased assessment of the study endpoint; Ⅵ, follow-up period appropriate to the aim of the study; Ⅶ, loss of follow up less than 5%; Ⅷ,prospective calculation of the study size.
