# Supplementary material for: Tyrosine kinase inhibitors in HER2‐positive breast cancer brain metastases: A systematic review and meta‐analysis
Source: Cancer Med. 2023 May 31;12(14):15090–100. doi: 10.1002/cam4.6180 (PMC10417165; doi:10.1002/cam4.6180)

**Figure S1.** Pooled median progression-free survival (PFS), median overall survival (OS) in TKI-containing regimens for HER2-positive breast cancer brain metastases: (A) Progression-free Survival (PFS); (B) Overall Survival (OS)


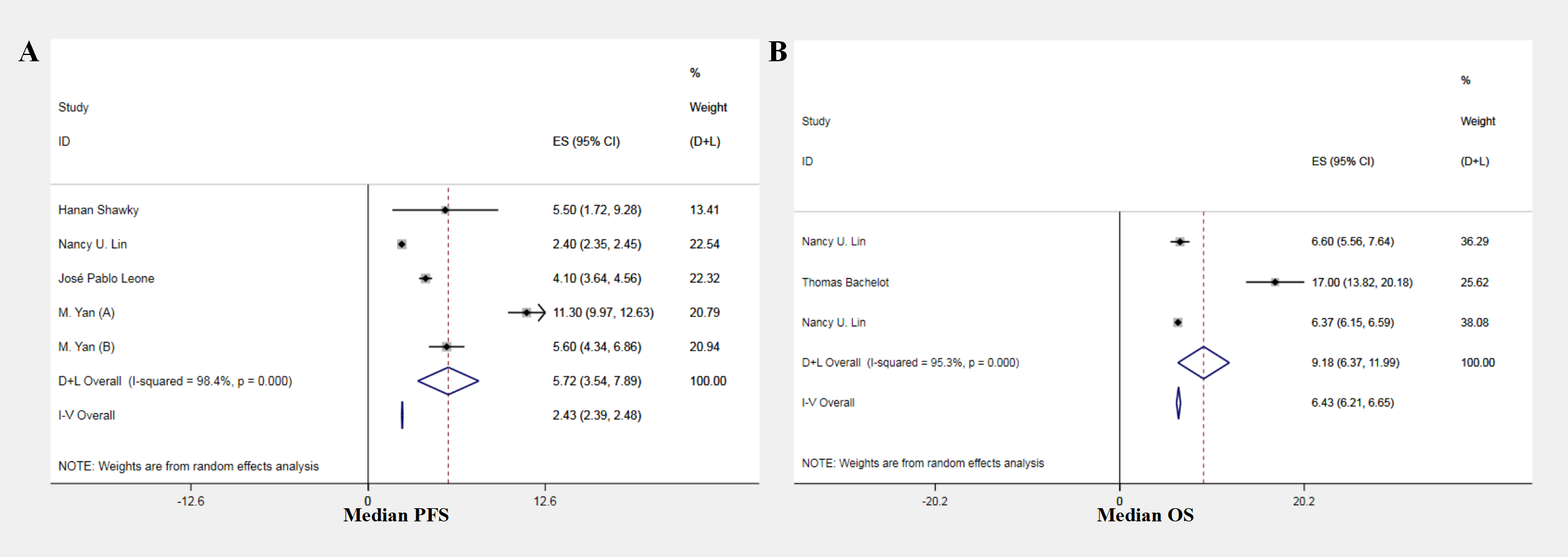

Supplement: Supplementary file 4 — Figure S1 [file CAM4-12-15090-s001.doc]
