# Supplementary figures and images for: Tyrosine kinase inhibitors in HER2‐positive breast cancer brain metastases: A systematic review and meta‐analysis
Source: Cancer Med. 2023 May 31;12(14):15090–100. doi: 10.1002/cam4.6180 (PMC10417165; doi:10.1002/cam4.6180)

**Figure S2.** Funnel plots of progression-free survival (PFS)


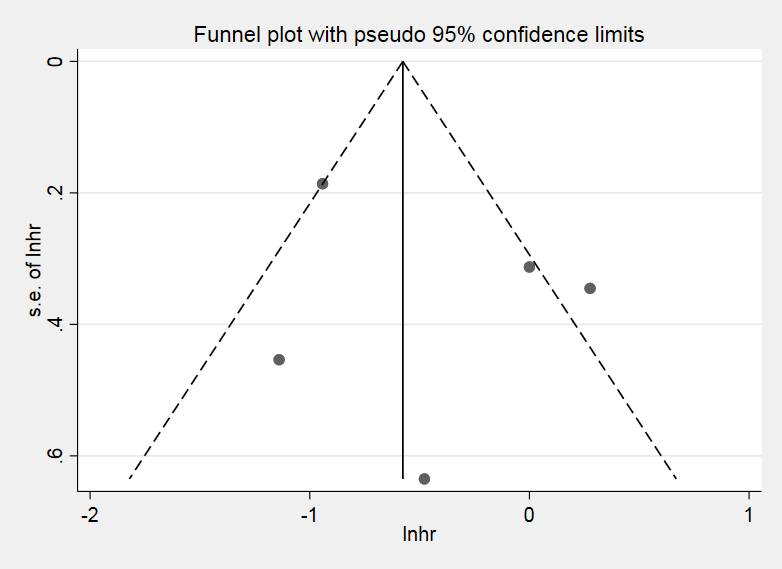

Supplement: Supplementary file 5 — Figure S2 [file CAM4-12-15090-s005.docx]
