# Supplementary material for: Tyrosine kinase inhibitors in HER2‐positive breast cancer brain metastases: A systematic review and meta‐analysis
Source: Cancer Med. 2023 May 31;12(14):15090–100. doi: 10.1002/cam4.6180 (PMC10417165; doi:10.1002/cam4.6180)
Supplement: Supplementary file 6 — Figure S3 [file CAM4-12-15090-s004.doc]

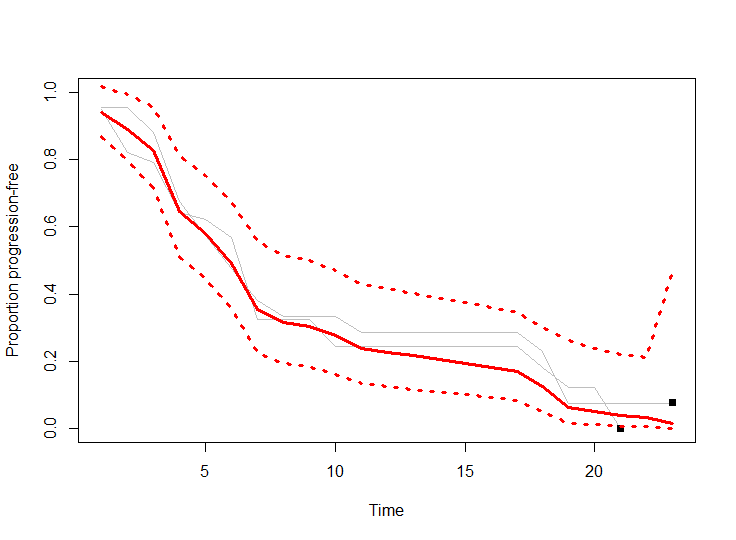


**A**


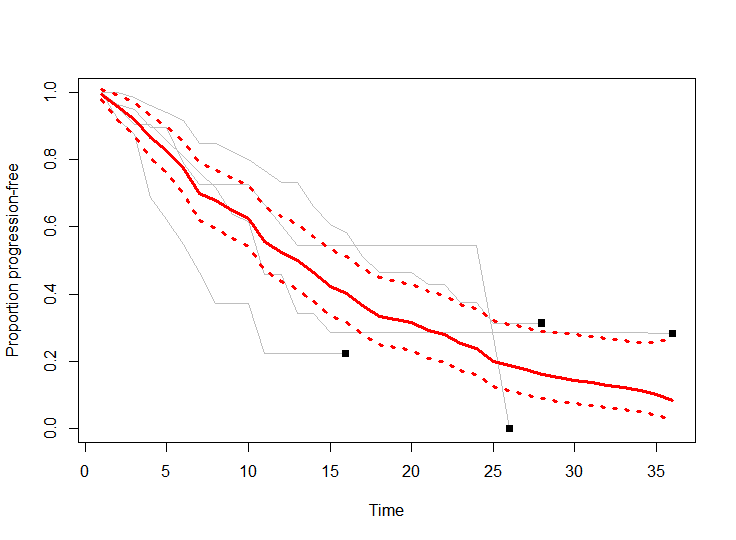


**B**

**Figure S3.** Progression-free survival (PFS) and Overall survival (OS) in Lapatinib-containing regimens for HER2-positive breast cancer and brain metastases

(A) Curves of the summary progression-free survival (PFS) for in Lapatinib-containing regimens for HER2-positive breast cancer and brain metastases in the 2 studies of the meta-analysis, Median PFS: 5.90 months (95% CI: 3.92-6.97); (B) Curves of the summary overall survival (OS) for in Lapatinib-containing regimens for HER2-positive breast cancer and brain metastases in the 4 studies of the meta-analysis, Median OS 12.99 months (95%CI: 10.30-14.92 ).

**Notes:** The gray lines represent the survival in each study, and the black square represents the end of follow-up. The thick lines represent the summarized survival curves with 95% confidence intervals (dashed lines) obtained using MetaSurv with a fixed-effects model.
